# Biomolecular Component Analysis of Phospholipids Composition in Live HeLa Cells

**DOI:** 10.3390/bios8040123

**Published:** 2018-12-05

**Authors:** Svitlana M. Levchenko, Junle Qu

**Affiliations:** Key Laboratory of Optoelectronic Devices and Systems of Ministry of Education and Guangdong Province, College of Optoelectronic Engineering, Shenzhen University, Shenzhen 518060, China; sveta.levchenko@gmail.com

**Keywords:** BCA, Raman microspectrometry, phospholipids, fatty acids, HeLa cells

## Abstract

The alteration of the phospholipid composition within the cell, in particular the ratio between saturated and unsaturated fatty acids, can serve as an important biomarker to prognosis of the disease progression (e.g., fatty-liver disease, prostate cancer, or neurodegenerative disorders). Major techniques for lipid analysis in biological samples require a lipid extraction procedure that is not compatible with live cell studies. To address this challenge, we apply microRaman-Biomolecular Component Analysis (BCA) for comparative analysis of phospholipid composition and sensing the saturation degree of fatty acid lipid chain in live HeLa cells and lipids extracted from HeLa cells. After processing raw Raman data, acquired in lipid droplets (LDs) free cytoplasmic area, LDs and extracted lipids with BCA, the lipid component was isolated. Despite the similarity in general profiles of processed Raman spectra acquired in live cells and extracted lipids, some clear differences that reflect diversity in their phospholipids composition were revealed. Furthermore, using the direct relation between the number of double bonds in the fatty acid chain and the intensity ratio of the corresponding Raman bands, the saturation degree of fatty acids was estimated.

## 1. Introduction

It is known that lipids play vital roles in establishing cellular architecture and maintaining cellular processes, including but not limited to energy storage, signaling reactions, protection against some forms of cellular stress, and many others [1,2,3]. Furthermore, their dysfunction has been linked to many diseases, e.g., obesity, diabetes, fatty liver disease, autoimmunity, prostate cancer, and some of the neurodegenerative disorders [4,5,6,7,8,9,10]. For instance, it was demonstrated that changes in the degree of lipid saturation can alter membrane fluidity, cell growth, and resistance to chemotherapeutic drugs [11,12]. Cancer cells can promote saturation of their membranes and modulate their biophysical properties and in this way protect themselves from lipid peroxidation-mediated cell death [12]. Also, the role of unsaturated fatty acids in protection of breast cancer cells from nutrient and lipotoxic stress was demonstrated [13]. It was also reported by other groups that the increasing amount of saturated fatty acids within the liver provokes endoplasmic reticulum stress, apoptosis, and liver injury [14,15]. Therefore, nowadays, fatty acid metabolic pathways are often considered as a target for anticancer therapy [16,17]. Moreover, alteration of polyunsaturated fatty acid levels and the signaling pathways that they regulate also was detected in various neurological disorders (e.g., Alzheimer’s disease and major depression) [4,5]. The relationship between the degree of phospholipid unsaturation and size of lipid droplets (LDs) was shown. The authors suggest that saturated fatty acid chains in phospholipid monolayers might establish the form and/or stability of large LDs that can contribute to the development of neurodegenerative disease [18,19]. Hence, the degree of phospholipids saturation cannot be ignored as it plays an important role in a wide range of intracellular processes and is often associated with various health issues [4,18,20].

These evidences evoke increasing interest in the development of analytical techniques for lipid analysis. Currently, a number of advanced techniques are frequently used as tools for chemically selective imaging or quantitative analysis of lipid content in biological samples [21,22,23,24,25]. A central analytical role in lipidomics belongs to different types of mass spectrometry (MS) that typically start with extraction of the lipids from biological samples [25,26,27,28]. As a result of the sample preparation procedure, information about cellular dynamics and functionality is often lost. Therefore, despite of numerous advantages (e.g., high sensitivity, relatively simple and fast analysis), these methods cannot be efficiently applied in live cell studies. Moreover, despite the ability of these approaches to provide detailed information about lipid content in biological samples, some important data is still missing, such as chain length or degree of saturation. Hence, for extraction of information about the level of phospholipids saturation, a more relevant approach is required.

From this perspective, microRaman-BCA approach offers an attractive alternative for analysis of phospholipid composition in a label-free and nondestructive way at the single-cell level without lipid extraction procedure required for most methods in lipidomics. The effectiveness of the micro-Raman-BCA assay for quantification of major classes of biomolecules, including lipids, in different organelles of live cells was demonstrated a number of times [10,29,30,31,32,33]. Particularly, comparative analysis of protein, RNA, and lipid concentrations in nucleoli, endoplasmic reticulum, and mitochondria of diploid lung fibroblasts and cancer cells was performed. The significant differences between the concentration profiles of normal diploid and cancer cell lines were found using combination of micro-Raman microscopy and BCA [29]. This method also can be successfully used for quantitative analysis of cellular heterogeneity [30]. Moreover, it was shown that lipid quantification by vibrational Raman Microspectroscopy and BCA may serve as a potential biomarker in prostate cancer [10].

In this study, we employed a microRaman-BCA approach for comparative analysis of phospholipids composition in live HeLa cells and lipids that were extracted from HeLa cells. The raw Raman spectra acquired in live cells are very complex and contain a lot of bands associated with different macromolecules that often overlap or partially overlap. Therefore, it can be very challenging to analyze and interpret the obtained results. Here, we demonstrated the analytical capability of the BCA approach to analyze the phospholipids composition from the Raman spectra acquired from LDs’ free cytoplasmic area, LDs of live cells, and lipids extracted from HeLa cells.

## 2. Materials and Methods

*Cell culture and fluorescence staining*. HeLa cells were plated onto glass bottom dishes (Mattek Ashland, MA, USA). HeLa cells were cultured in advanced DMEM (Life Technologies, Carlsbad, CA, USA), supplemented with 2.5% fetal calf serum (FBS) (Sigma, St. Louis, MO, USA), 1% glutamax (Life Technologies), and 1% antibiotic antimycotic solution (Sigma) at 37 °C in a humidified atmosphere, containing 5% CO_2_.

*Extraction of lipid from HeLa cells*. The total lipids were extracted directly from the cell dishes using ethanol. The extracted lipids were transferred to another glass dish and dried. The dried lipids were further analyzed.

*Raman microspectrometry*. The confocal Raman microspectrometer system consists of an inverted Nikon TE200 microscope equipped with single frequency laser diode (Ondax, Monrovia, CA, USA 638 nm, 120 mW) excitation source, fiber-input MS3501i imaging monochromator/spectrograph (Solar TII, Minsk, Republic of Belarus), and HS101H−2048/122-HR2 series CCD (Proscan, SOL instruments Ltd., Minsk, Republic of Belarus) cooled down to −30 °C. The spectral resolution for the fixed diffraction grating position (wavenumber interval between ~580 and 1800 cm^−1^) was ~1.5 cm^−1^. The excitation laser beam from a single frequency laser diode (Ondax, 638 nm) of power ~70 mW was focused onto the sample in a spot of diameter ~0.8 μm, using a 100× Nikon oil-immersion objective lens with NA = 1.3. A 100 μm pinhole provides for confocal acquisition of the Raman signal, which corresponded to a confocal parameter of ~1.8 μm (in FWHM for λ = 638 nm). A series of three Raman spectra were acquired with an accumulation time of 60 s per each spectrum and then averaged, giving a total spectral integration time of 180 s per measurement.

*Biomolecular Component Analysis*. The lipid component was isolated and analyzed, as described in detail in previous publications [31,32,33]. Briefly, the spectra were subsequently processed by a BCA algorithm using BCAbox (ACIS, LLC, Buffalo, NY, USA), which includes background subtraction, baseline correction, and nonlinear least squares curve fitting to identify and quantify the contributions made by RNA, DNA, lipids, and proteins. The corresponding biomolecular components serves as a model component for background subtraction and isolation of spectral contribution for lipids are illustrated in Figure S1A,B. The simplified algorithm of background and baseline subtraction together with Raman profiles of background components provided by the software developer are illustrated in Figure S1B–D. After preprocessing (Figure S2A), the weighted spectra of RNA, DNA, and protein components were subtracted from measured spectra (Figures S2B and S4). Corresponding BCA coefficients for HeLa Raman spectrum are shown in Table S1. The residual spectra belonging to lipids were normalized to the intensity of the peak at 1440 cm^−1^. These lipid components were used for subsequent analysis.

## 3. Results and Discussion

During the Raman measurements, intracellular LDs and the cytoplasm without LDs inclusions were identified by their characteristic dense appearance in the transmitted light. Aimed to figure out the difference in phospholipid composition between different sites in live cells as well as to explore the impact of extraction of lipids procedure on phospholipids content compared with live cells, the Raman data from various sites in live cells and extracted lipids were processed and analyzed.

**Figure 1:**
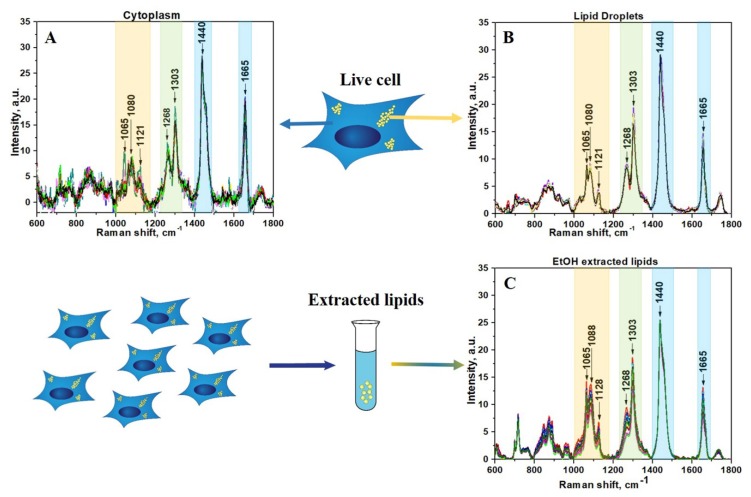
(**A**) Lipids component of cytoplasm of live HeLa cells; (**B**) Lipids component of LDs in live HeLa cells; (**C**) Lipids component of EtOH extracted lipids from HeLa cells. The wavenumber regions assigned to fatty acids are shown as colored bands.

Raman spectra were acquired in different cells from 28 different areas in cytoplasm, 22 areas in LDs, and 12 areas from lipid extract. After applying preprocessing routine and BCA to raw Raman data, the lipid component was isolated using previously established techniques (Figure 1).

**Figure 2:**
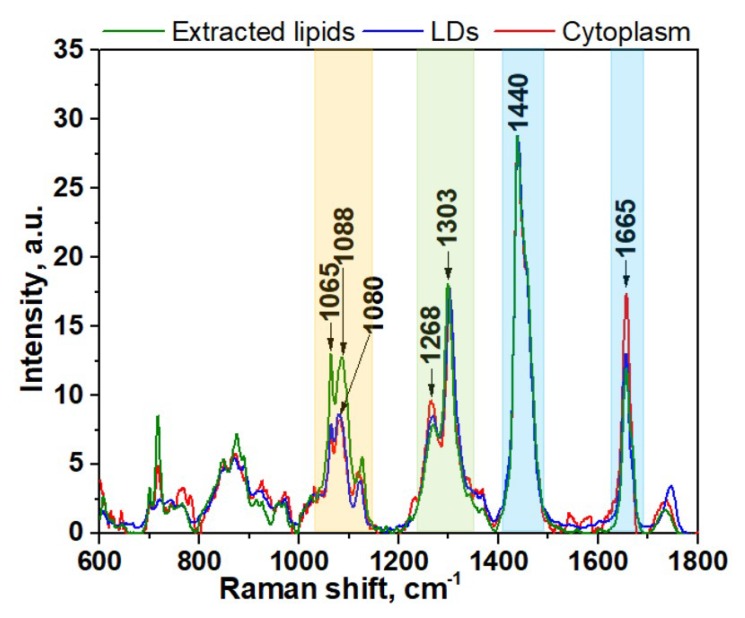
Averaged spectra of lipids component of cytoplasm, LDs, and extracted lipids of HeLa cells. The wavenumber regions assigned to fatty acids are shown as colored bands.

The spectral region of fatty acids can be recognized by group of bands in the region between 1000 and 1200 cm^−1^ (Figure 1, yellow color) due to C–C stretching vibrations and the prominent bands at about 1268 and 1303 cm^−1^ (Figure 1, green color) due to =C–H cis stretches and C–H_2_ twist vibrations, 1440 and 1665 cm^−1^ (Figure 1, blue color) due to C–H_2_ bending and C=C stretching mode in unsaturated fatty acids, correspondingly [34,35,36,37]. Although the general profile of the processed Raman spectra of lipids obtained for cytoplasm is quite similar to the spectra of LDs or extracted lipids, there are some clear differences that reflect diversity in their phospholipids composition (Figure 1A,B). In order to make these differences more evident, the averaged Raman spectra for lipid component of cytoplasm, LDs, and extracted lipids were obtained and further comparatively analyzed (Figure 2). Specifically, the band at 1080 cm^−1^ in the spectra of extracted lipids was shifted toward a higher wavenumber of 1088 cm^−1^ compared with spectra from live cells. Previously, it was demonstrated that fatty acid chain elongation leads to the shift of the C–C stretch vibration band to the longer wavenumbers [34]. Therefore, this is most probably associated with the prevailing of fatty acids with longer chain lengths in extracted lipids than in lipids of live cells.

Further comparison of the Raman spectra also revealed pronounced differences in intensity profiles of studied spectra. For instance, in the spectra of the extracted lipids and LDs, the relative intensity of the band at 1268 cm^−1^ assigned to =C–H stretching is lower than in the spectra of cytoplasm (Figure 2, green colored band). The intensity of the three bands in the low frequency region (1000–1200cm^−1^) of fatty acids are significantly higher for extracted lipids (Figure 2, yellow colored band). Also, the intensity of the C=C stretching band at 1655 cm^−1^ is significantly increased in the spectra of cytoplasm compared to LDs and extracted lipids. Altogether, these data suggest that the cytoplasm of live cells typically contains more double bonds and consequently a higher percentage of unsaturated fatty acids than LDs and extracted lipids. Based on these facts, we can assume that the phospholipid composition of LDs in live cells and lipids extracted from HeLa cells are similar, while they noticeably differ from cytoplasmic lipid content. This also allows us to conclude that the lipid extraction procedure results in noticeable change of fatty acids composition compared with live cells. It should be pointed out that often their types may be more important than their quantity with regard to health and disease.

For the next step, the processed spectra were used to determine the saturation degree of intracellular fatty acids in different areas of cytoplasm and extracted lipids from cells. Information about the degree of saturation of fatty acids can be obtained from the ratio of Raman intensity of specific bands that correspond to the C=C stretch and C–H_2_ bend, correspondingly [35,38,39,40]. There is a direct link between number of double bonds in the fatty acid chain and the intensity ratio of the specific bands at 1665 cm^−1^ (stretching mode proportional to the amount of unsaturated C=C bonds) and 1440 cm^−1^ (C–H_2_ bending mode proportional to the amount of saturated C–C bonds).

**Figure 3:**
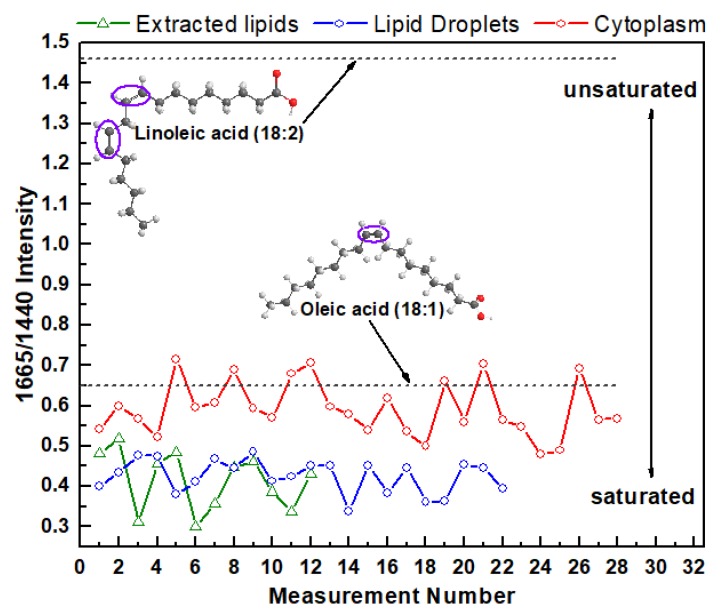
I(1665)/I(1440) Raman intensity ratio of the lipids from live HeLa cells (cytoplasm and LDs) and extracted lipids.

The Raman ratio I(1665)/I(1440) for every measurement from the lipid extracted from HeLa cells and from the lipids in cytoplasm and LDs in live cells are plotted on the same chart (Figure 3). As a reference, this intensity ratio was also determined and plotted on the same chart for monosaturated oleic acids that contain one C=C bond and polyunsaturated linoleic acids that contain two C=C bonds. For oleic and linoleic acids these ratios are 0.65 and 1.46 [40], respectively (Figure 3). The higher ratio I(1665)/I(1440) corresponds to the higher degree of lipid-chain unsaturation.

These results demonstrate that the degree of intracellular fatty acid saturation varies significantly not only in cytoplasmic LDs and LDs free cytoplasmic area, but also vary from one measurement to another. For example, the Raman intensity ratio varies from 0.48 to 0.72 for LDs free cytoplasmic area, from 0.34 to 0.49 for LDs, and from 0.30 to 0.51 for extracted lipids. Interestingly, on average, the variation in degree of saturation in LDs free cytoplasmic area is more diverse compared with the variation in different LDs. Moreover, this data suggests that cellular cytoplasm contain higher relative unsaturated lipid concentrations than in LDs or extracted lipids. Specifically, the average value of I(1665)/I(1440) for cytoplasm is 0.59 ± 0.06 (mean ± s.d.) while it is 0.42 ± 0.04 for LDs and 0.41 ± 0.06 for extracted lipids. These data also revealed that the degree of saturation of fatty acids in LDs and extracted lipids is almost the same. This finding reflects the fact that the EtOH lipid extraction procedure leads to preferential LDs lipids preservation, while the major fraction of cytoplasmic lipids is washed out from the extract or remains in the cell.

## 4. Conclusions

A comparative analysis of processed Raman spectra of LDs, LDs free cytoplasmic area, and extracted lipids revealed that phospholipid content is significantly different in live cells and extracted lipids. Although the extracted lipids show all major lipid Raman bands, which are also present in the spectra measured in live cells, the BCA approach revealed that the lipid extraction procedure results in noticeable changes in fatty acid composition compared with live cells. In particular, it was shown that the degree of intracellular fatty acids saturation differs significantly for LDs and LDs free cytoplasmic area and varies between measurements. Furthermore, this data demonstrates that the cellular cytoplasm contains higher relative unsaturated lipid concentrations (0.59 ± 0.06) than in LDs (0.42 ± 0.04) or extracted lipids (0.41 ± 0.06). Altogether, these findings demonstrate that in contrast to traditional lipidomics approaches which involve cell destruction with subsequent biochemical analysis, the microRaman-BCA approach is capable of performing noninvasive analysis of phospholipid composition in situ. This capability expands the list of available analytical tools in the emerging field of lipidomics for multiple applications.

## Supplementary Materials

The following are available online at http://www.mdpi.com/2079-6374/8/4/123/s1, Figure S1: (A) Raman spectrum of the DNA, lipids, proteins and RNA that were used as the reference spectra for the BCA analysis^1^. Unit weight of each biomolecular component was chosen to correspond to 100 mg/ml for proteins and 20 mg/ml for RNA, DNA and lipids concentrations. (B) Raman profiles of background components and representative background component with weighted coefficients. (C) Simplified algorithm of background subtraction from raw Raman spectra provided by software developer BCAbox (ACIS, LLC, Buffalo, NY, USA), Figure S2: (A) Representative raw Raman spectra of cytoplasm in HeLa cell and background profile with corresponding weighted coefficients. (B) Corresponding preprocessed free background spectrum, residual spectrum and lipids component obtained after subtraction of the DNA, RNA and proteins weighted model components from the preprocessed spectrum, Figure S3: The input model spectrum of lipid component (blue), model spectrum of lipid component obtained for specific measurement after BCA (red), lipid component obtained after subtraction of the weighted spectra of RNA, DNA, and protein components from measured spectra (black), Figure S4: Raw (black, upper curve) and pre-processed (red lower curve) Raman spectra of cytoplasm area in HeLa cell (upper panel). Lower panel illustrates residual spectra after subtraction of model spectra of cell and background from cell part (black) and background part of preprocessed spectrum shown in upper panel, Table S1: BCA coefficients for HeLa Raman spectrum, shown in Figure S4. Error was calculated on the basis of residual spectra, shown in lower panel of figure. LSU is lipids unsaturation parameter.

## References

[1] Welte M.A., Gould A.P. (2017). Lipid droplet functions beyond energy storage. Biochim. Biophys. Acta.

[2] Bailey A.P., Koster G., Guillermier C., Hirst E.M., MacRae J.I., Lechene C.P., Postle A.D., Gould A.P. (2015). Antioxidant role for lipid droplets in a stem cell niche of drosophila. Cell.

[3] Lizardo D.Y., Parisi L.R., Li N., Atilla-Gokcumen G.E. (2018). Noncanonical roles of lipids in different cellular fates. Biochemistry.

[4] Bazinet R.P., Laye S. (2014). Polyunsaturated fatty acids and their metabolites in brain function and disease. Nat. Rev. Neurosci..

[5] Sato N., Morishita R. (2015). The roles of lipid and glucose metabolism in modulation of beta-amyloid, tau, and neurodegeneration in the pathogenesis of alzheimer disease. Front. Aging Neurosci..

[6] Aufschnaiter A., Kohler V., Diessl J., Peselj C., Carmona-Gutierrez D., Keller W., Buttner S. (2017). Mitochondrial lipids in neurodegeneration. Cell Tissue Res..

[7] Peirce V., Carobbio S., Vidal-Puig A. (2014). The different shades of fat. Nature.

[8] Samuel V.T., Shulman G.I. (2018). Nonalcoholic fatty liver disease as a nexus of metabolic and hepatic diseases. Cell Metab..

[9] Serhan C.N. (2014). Pro-resolving lipid mediators are leads for resolution physiology. Nature.

[10] O’Malley J., Kumar R., Kuzmin A.N., Pliss A., Yadav N., Balachandar S., Wang J., Attwood K., Prasad P.N., Chandra D. (2017). Lipid quantification by raman microspectroscopy as a potential biomarker in prostate cancer. Cancer Lett..

[11] Deep G., Schlaepfer I.R. (2016). Aberrant lipid metabolism promotes prostate cancer: Role in cell survival under hypoxia and extracellular vesicles biogenesis. Int. J. Mol. Sci..

[12] Rysman E., Brusselmans K., Scheys K., Timmermans L., Derua R., Munck S., Van Veldhoven P.P., Waltregny D., Daniels V.W., Machiels J. (2010). De novo lipogenesis protects cancer cells from free radicals and chemotherapeutics by promoting membrane lipid saturation. Cancer Res..

[13] Jarc E., Kump A., Malavasic P., Eichmann T.O., Zimmermann R., Petan T. (2018). Lipid droplets induced by secreted phospholipase a2 and unsaturated fatty acids protect breast cancer cells from nutrient and lipotoxic stress. Biochim. Biophys. Acta.

[14] Wang D., Wei Y., Pagliassotti M.J. (2006). Saturated fatty acids promote endoplasmic reticulum stress and liver injury in rats with hepatic steatosis. Endocrinology.

[15] Wei Y., Wang D., Gentile C.L., Pagliassotti M.J. (2009). Reduced endoplasmic reticulum luminal calcium links saturated fatty acid-mediated endoplasmic reticulum stress and cell death in liver cells. Mol. Cell. Biochem..

[16] Chen T., Li H. (2017). Fatty acid metabolism and prospects for targeted therapy of cancer. Eur. J. Lipid Sci. Technol..

[17] Liu Q., Luo Q., Halim A., Song G. (2017). Targeting lipid metabolism of cancer cells: A promising therapeutic strategy for cancer. Cancer Lett..

[18] Arisawa K., Mitsudome H., Yoshida K., Sugimoto S., Ishikawa T., Fujiwara Y., Ichi I. (2016). Saturated fatty acid in the phospholipid monolayer contributes to the formation of large lipid droplets. Biochem. Biophys. Res. Commun..

[19] Liu L., Zhang K., Sandoval H., Yamamoto S., Jaiswal M., Sanz E., Li Z., Hui J., Graham B.H., Quintana A. (2015). Glial lipid droplets and ros induced by mitochondrial defects promote neurodegeneration. Cell.

[20] Dowhan W. (1997). Molecular basis for membrane phospholipid diversity: Why are there so many lipids?. Annu. Rev. Biochem..

[21] Le T.T., Yue S., Cheng J.-X. (2010). Shedding new light on lipid biology with coherent anti-stokes raman scattering microscopy. J. Lipid Res..

[22] Syed A., Smith E.A. (2017). Raman imaging in cell membranes, lipid-rich organelles, and lipid bilayers. Annu. Rev. Anal. Chem..

[23] Quehenberger O., Armando A.M., Dennis E.A. (2011). High sensitivity quantitative lipidomics analysis of fatty acids in biological samples by gas chromatography-mass spectrometry. Biochim. Biophys. Acta.

[24] Park H.M., Shon J.C., Lee M.Y., Liu K.H., Kim J.K., Lee S.J., Lee C.H. (2014). Mass spectrometry-based metabolite profiling in the mouse liver following exposure to ultraviolet b radiation. PLoS ONE.

[25] Harkewicz R., Dennis E.A. (2011). Applications of mass spectrometry to lipids and membranes. Annu. Rev. Biochem..

[26] Cajka T., Fiehn O. (2014). Comprehensive analysis of lipids in biological systems by liquid chromatography-mass spectrometry. TrAC Trends Anal. Chem..

[27] Murphy R.C., Gaskell S.J. (2011). New applications of mass spectrometry in lipid analysis. J. Biol. Chem..

[28] Kim H.Y., Lee H., Kim S.H., Jin H., Bae J., Choi H.K. (2017). Discovery of potential biomarkers in human melanoma cells with different metastatic potential by metabolic and lipidomic profiling. Sci. Rep..

[29] Levchenko S.M., Kuzmin A.N., Pliss A., Qu J., Prasad P.N. (2017). Macromolecular profiling of organelles in normal diploid and cancer cells. Anal. Chem..

[30] Kuzmin A.N., Levchenko S.M., Pliss A., Qu J., Prasad P.N. (2017). Molecular profiling of single organelles for quantitative analysis of cellular heterogeneity. Sci. Rep..

[31] Kuzmin A.N., Pliss A., Kachynski A.V. (2013). Biomolecular component analysis of cultured cell nucleoli by raman microspectrometry. J. Raman Spectrosc..

[32] Kuzmin A.N., Pliss A., Prasad P.N. (2014). Changes in biomolecular profile in a single nucleolus during cell fixation. Anal. Chem..

[33] Kuzmin A.N., Pliss A., Prasad P.N. (2017). Ramanomics: New omics disciplines using micro raman spectrometry with biomolecular component analysis for molecular profiling of biological structures. Biosensors.

[34] De Gelder J., De Gussem K., Vandenabeele P., Moens L. (2007). Reference database of raman spectra of biological molecules. J. Raman Spectrosc..

[35] Wu H., Volponi J.V., Oliver A.E., Parikh A.N., Simmons B.A., Singh S. (2011). In vivo lipidomics using single-cell raman spectroscopy. Proc. Natl. Acad. Sci. USA.

[36] Schie I.W., Huser T. (2013). Methods and applications of raman microspectroscopy to single-cell analysis. Appl. Spectrosc..

[37] Zinin P.V., Misra A., Kamemoto L., Yu Q.G., Hu N.J., Sharma S.K. (2010). Visible, near-infrared, and ultraviolet laser-excited raman spectroscopy of the monocytes/macrophages (U937) cells. J. Raman Spectrosc..

[38] Munchberg U., Wagner L., Rohrer C., Voigt K., Rosch P., Jahreis G., Popp J. (2015). Quantitative assessment of the degree of lipid unsaturation in intact mortierella by raman microspectroscopy. Anal. Bioanal. Chem..

[39] Le T.T., Duren H.M., Slipchenko M.N., Hu C.D., Cheng J.X. (2010). Label-free quantitative analysis of lipid metabolism in living caenorhabditis elegans. J. Lipid Res..

[40] Samek O., Jonas A., Pilat Z., Zemanek P., Nedbal L., Triska J., Kotas P., Trtilek M. (2010). Raman microspectroscopy of individual algal cells: Sensing unsaturation of storage lipids in vivo. Sensors.

