# Supplementary material for: Biomolecular Component Analysis of Phospholipids Composition in Live HeLa Cells
Source: Biosensors (Basel). 2018 Dec 5;8(4):123. doi: 10.3390/bios8040123 (PMC6315881; doi:10.3390/bios8040123)
Supplement: Supplementary file 1 [file biosensors-08-00123-s001.pdf]

Supporting Information

Biomolecular Component Analysis of Phospholipids composition in live HeLa cells

Svitlana M. Levchenko[1], Junle Qu[1*]

[1] Key Laboratory of Optoelectronic Devices and Systems of Ministry of Education and Guangdong Province, College of Optoelectronic Engineering, Shenzhen University, Shenzhen, Guangdong Province 518060, China

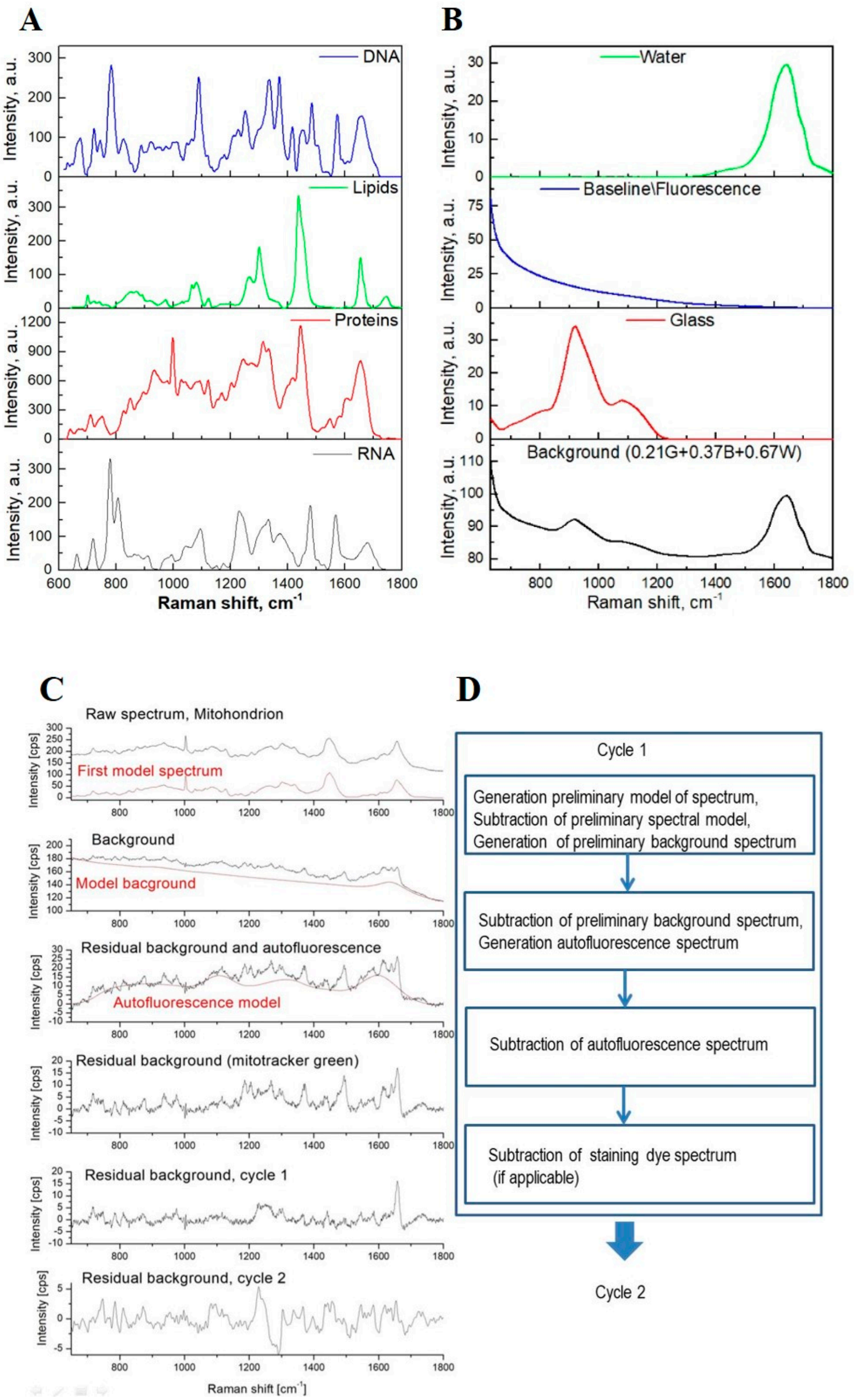

Figure S1. (A) Raman spectrum of the DNA, lipids, proteins and RNA that were used as the reference spectra for the BCA analysis[1]. Unit weight of each biomolecular component was chosen to correspond to 100 mg/ml for proteins and 20 mg/ml for RNA, DNA and lipids concentrations. (B) Raman profiles of background components and representative background component with weighted coefficients. (C) Simplified algorithm of background subtraction from raw Raman spectra provided by software developer BCAbox (ACIS,LLC, Buffalo, NY, USA).

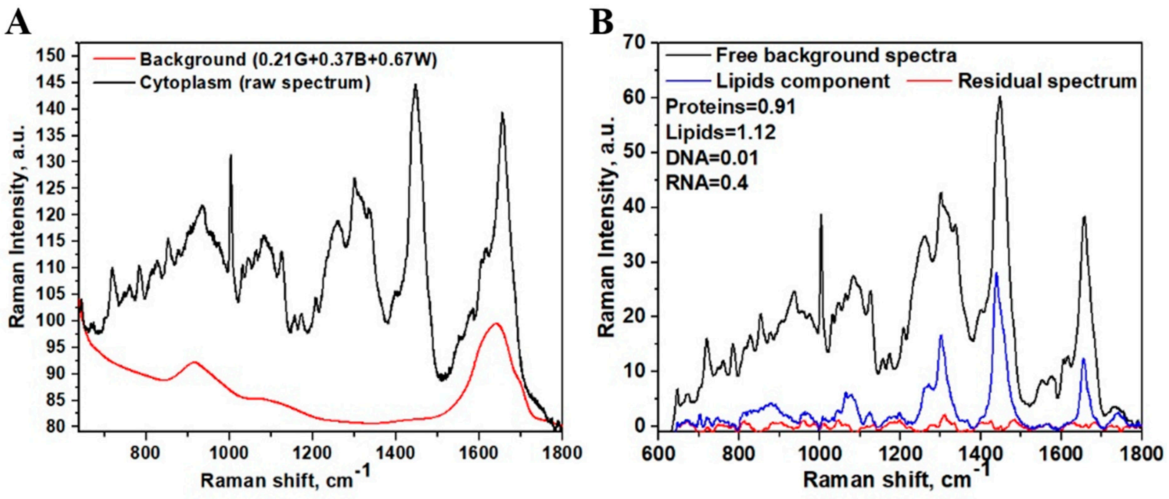

Figure S2. (A) Representative raw Raman spectra of cytoplasm in HeLa cell and background profile with corresponding weighted coefficients. (B) Corresponding preprocessed free background spectrum, residual spectrum and lipids component obtained after subtraction of the DNA, RNA and proteins weighted model components from the preprocessed spectrum.

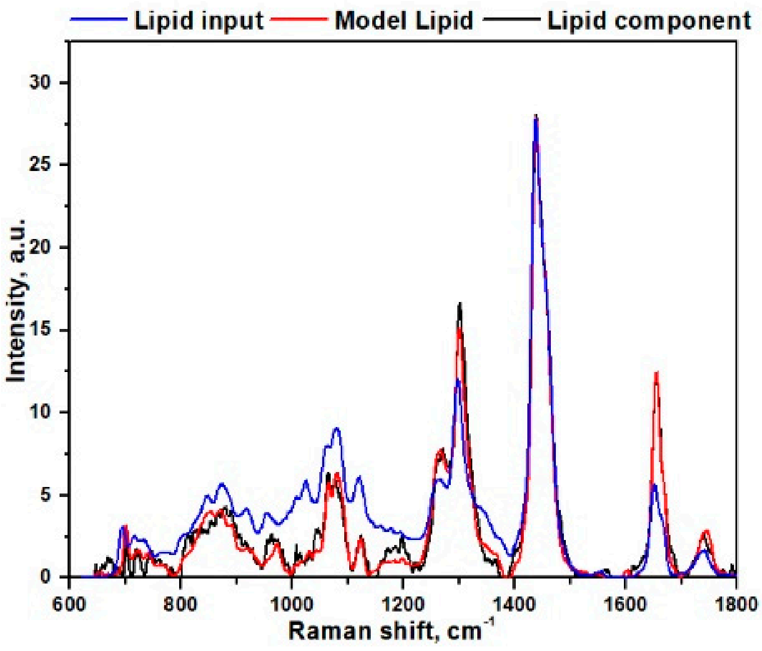

Figure S3. The input model spectrum of lipid component (blue), model spectrum of lipid component obtained for specific measurement after BCA (red), lipid component obtained after subtraction of the weighted spectra of RNA, DNA, and protein components from measured spectra (black).

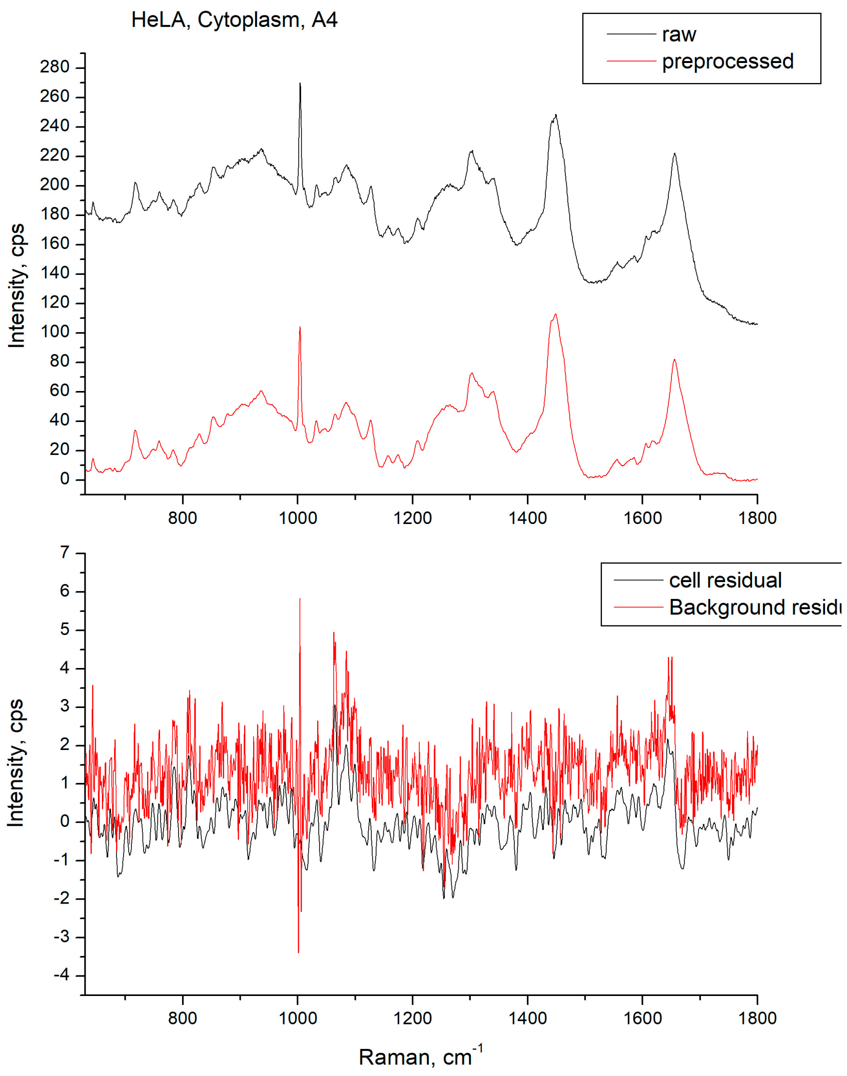

Figure S4. Raw (black, upper curve) and pre-processed (red lower curve) Raman spectra of cytoplasm area in HeLa cell (upper panel). Lower panel illustrates residual spectra after subtraction of model spectra of cell and background from cell part (black) and background part of preprocessed spectrum shown in upper panel.

**Parameter**	**Weight**	**Error (+/-)**
Proteins	1.27	0.05
DNA	0.03	0.05
RNA	0.32	0.05
Lipids	1.92	0.05
Glycogen	0.03	0.05
LSU	0.47	
Glass	0	0.05
B/F	0.92	0.05
Water	0.72	0.05

Table S1. BCA coefficients for HeLa Raman spectrum, shown in Fig 4S. Error was calculated on the basis of residual spectra, shown in lower panel of figure. LSU is lipids unsaturation parameter.

References

1. Kuzmin, A. N.; Pliss, A.; Kachynski, A. V., Biomolecular component analysis of cultured cell nucleoli by Raman microspectrometry. *Journal of Raman Spectroscopy* **2013,** *44* (2), 198-204.
